# Genetic Variation of Chicken *Growth Differentiation Factor-9* Gene and Association With Egg Characteristics: A Systematic Review

**DOI:** 10.1155/tswj/2659937

**Published:** 2026-06-27

**Authors:** Lehlogonolo Joel Morumbula Maake, Salome Mhlabini, Travor Tshepiso Magonyane, Raisibe Lisbert Mahlo, Madumetja Cyril Mathapo, Thobela Loius Tyasi

**Affiliations:** ^1^ Department of Agricultural Economics and Animal Production, University of Limpopo, Sovenga, South Africa, ul.ac.za

**Keywords:** egg quality, *GDF-9* gene, layers, mutation, single nucleotide polymorphism

## Abstract

Chicken layers are valued for their ability to produce eggs, an essential component of the human diet. Egg characteristics are influenced by genetic factors such as the growth differentiation factor‐9 (GDF‐9) gene, which plays a vital role in follicular development and ovulation. Variations in the GDF‐9 gene can lead to ovulation dysfunction, infertility, and reduced egg quality and production. This systematic review explored the genetic variation of the chicken GDF‐9 gene and its association with egg characteristics. Following the Preferred Reporting Items for Systematic Reviews and Meta‐Analyses (PRISMA) guidelines, databases including ScienceDirect, Google Scholar, PubMed, and Web of Science were used to search for articles using combinations of keywords: “chickens,” “GDF‐9 gene,” “genetic variation,” and “egg characteristics.” A total of 106 articles were retrieved, and five met the inclusion criteria. The reviewed studies identified several single nucleotide polymorphisms (SNPs) within the GDF‐9 gene—namely g.171515C>T, g.171530A>G, g.171529G>A, g.171554C>T, g.171577A>G, g.171600C>T, g.171541A>G, g.171560T>C, and g.171589C>T. Although no identical SNPs were shared among all breeds, these variants were associated with key egg traits such as age at first egg, egg number, and egg weight. The findings suggest that GDF‐9 gene polymorphisms hold strong potential for use in marker‐assisted and genomic selection to enhance reproductive performance and egg‐laying efficiency in chickens. However, the current findings are primarily based on conventional genetic association studies, and higher resolution transcriptomic approaches, such as single‐cell RNA sequencing, are needed to functionally validate these associations and clarify the molecular mechanisms underlying GDF‐9 regulation in reproductive traits. Further research using genetically diverse populations and incorporating gene expression analysis is recommended to validate these associations and strengthen the application of GDF‐9 in poultry breeding.

## 1. Introduction

The genetic improvement of layers plays a crucial role in enhancing productivity and efficiency in egg production, given the growth of the human population [1]. Egg characteristics such as egg weight (EW), shell quality, yolk index, albumen index, Haugh unit, and chemical composition influence both economic profitability and consumer preferences [2]. Those egg characteristics are affected by some genetic factors, such as the *growth differentiation factor-9* gene (*GDF-9*) [3, 4]. The *GDF-9* gene serves a vital role in follicle development, which directly influences the reproductive traits in layers [4]. Research indicates that *GDF-9* gene mutations lead to ovulation dysfunction, infertility, and early ovarian failure, thus leading to poor egg characteristics and quantity [4]. Advances in molecular genetics, particularly genome‐wide association studies (GWAS), provide opportunities to identify single nucleotide polymorphisms (SNPs) associated with egg production traits [5]. Once favorable alleles are identified, marker‐assisted selection (MAS) can be employed to enhance breeding efficiency and reproductive performance [6]. Although studies by Liu et al. [4] and Huang et al. [7] have identified SNPs within the GDF‐9 gene and explored their association with reproductive traits, findings remain inconsistent across chicken breeds. According to our knowledge, there is no systematic review on the GDF‐9 genetic variations and their association with egg characteristics in chickens. Therefore, this review is aimed at systematically reviewing literature on the genetic variations of the chicken GDF‐9 gene and evaluating their association with egg characteristics. The findings will provide insight into the genetic basis of egg production, support the development of marker‐assisted breeding programs, and ultimately contribute to improving productivity and sustainability in the poultry industry.

## 2. Methods and Materials

### 2.1. Eligibility Criteria

Identification of the population, intervention, comparison, and outcomes (PICO) components of the research was performed for this systematic review, as described by Eriksen and Frandsen [8]. The “chickens” were defined as the population of the study, with the “*GDF-9* gene” as an intervention, “genetic variation” as the comparison, and “egg characteristics” as the outcome. Before conducting the study, a preliminary search of the PICO components on Google Scholar, ScienceDirect, PubMed, and Web of Science was conducted.

### 2.2. Search Strategy for Identification of Relevant Studies

The methodology for this review used Preferred Reporting Items for Systematic Reviews and Meta‐Analyses (PRISMA) by Sarkis‐Onofre et al. [9] when preparing the literature search for the review. Search databases such as Google Scholar, ScienceDirect, PubMed, and Web of Science were used to search for literature before the write‐up. The literature search for this systematic review included keywords such as chickens OR layers OR hens, AND “*GDF-9* gene,” AND “genetic variation” OR mutation OR SNPs OR “genetic diversity” OR polymorphism, AND “egg characteristics” OR “egg traits” OR “egg quality” OR “egg morphology.”

### 2.3. Inclusion Criteria

This systematic review included articles written in English and investigated the genetic variation of the chicken *GDF-9* gene and its association with egg characteristics.

### 2.4. Exclusion Criteria

The criteria for exclusion involved the following: duplicate articles, articles not written in English, and review articles. It also excluded articles that had only abstracts, articles that do not have keyword combinations, and the ones that use other animal species other than chicken.

### 2.5. Data Extraction

The data from the included articles was extracted by Maake Lehlogonolo Joel Morumbula and Thobela Loius Tyasi. The data included the first author′s name, year of publication, the country where the study was conducted, the journal that the article has been published, chicken breed, genetic variation of chicken *GDF-9* gene, and egg characteristics.

## 3. Results

### 3.1. Searched Results

**Figure 1:**
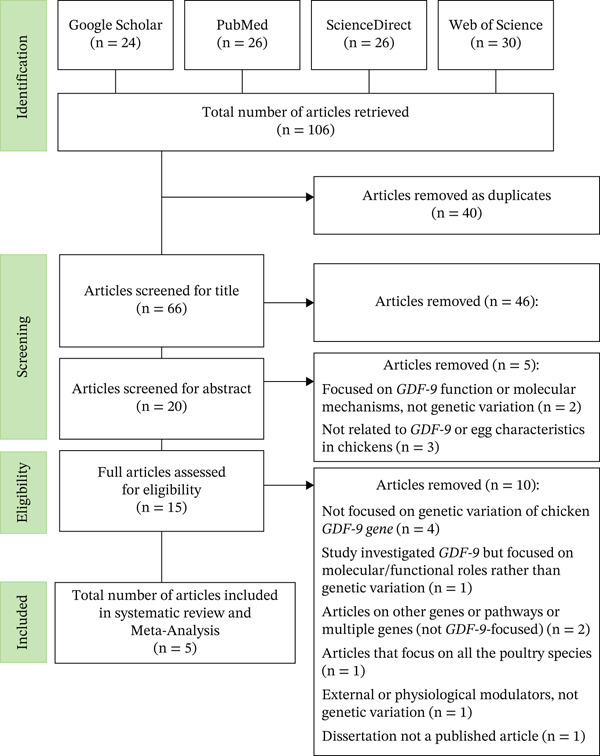
Flow chart of identification and selection of articles used in the systematic review.

A total of 106 articles (*n* = 106) were retrieved from search databases such as Google Scholar (*n* = 24), PudMed (*n* = 26), ScienceDirect (*n* = 26), and Web of Science (*n* = 30) as shown in Figure 1. Forty articles (*n* = 40), which occurred as duplicates, were removed. After screening for title, abstract, and eligibility, 61 articles (*n* = 61) were excluded. A total of five articles (*n* = 5) were included in the systematic review.

### 3.2. Characterization of Included Studies

Characterization of the five included articles (*n* = 5) are illustrated in Table 1. The results indicated that four articles (*n* = 4) investigated polymorphisms in the *GDF-9* gene in various Chinese indigenous breeds [4, 7, 11, 12]. Two articles (*n* = 2) investigated the contribution of other reproductive‐related genes, such as *FOXL2* [12] and *BMP-15* [7]. Only one article (*n* = 1) examined the levels of *GDF-9* gene expression in the oviduct tissues of Lohman Brown, Golden Sabahia, and White Leghorn chickens [10]. Liu et al. [4] had the largest chicken sample size (*n* = 511), whereas Habashy and Adomako [10] had the smallest (*n* = 268).

**Table 1:** Characterization of included studies.

Author	Year	Country	Breed	Sample size	*GDF-9* gene variation	Genotyping method
Habashy and Adomak [10]	2023	Egypt	Lohman Brown	96	Expression levels in oviduct tissues	RT‐qPCR (expression analysis)
			Golden Sabahia	102		
			White Leghorn	70		

Huang et al. [7]	2015	China	Shaobo hens	350	Polymorphisms in *GDF-9* and *BMP-15* gene*s*	PCR/PCR‐RFLP and DNA sequencing

Liu et al. [4]	2018	China	Dongxiang blue‐shelled	279	Polymorphisms in *GDF-9* gene	Direct DNA sequencing of PCR products; association analysis
			Luhua chickens	232		

Lou et al. [11]	2018	China	Jinghai Yellow	373	Polymorphisms in *GDF-9* gene	PCR‐SSCP screening followed by DNA sequencing

Qin et al. [12]	2015	China	Dagu hens	360	Polymorphisms in *GDF-9* and *FOXL2* genes	PCR‐SSCP and sequencing for SNP detection and genotyping

Abbreviations: *BMP-15*, *bone morphogenetic protein 15*; *FOXL2*, *forkhead box L2*; *GDF-9*, *growth differentiation factor*‐*9*; PCR, polymerase chain reaction; PCR‐SSCP, polymerase chain reaction–single‐strand conformation polymorphism; RFLP, restriction fragment length polymorphism; RT‐qPCR, reverse transcription–quantitative polymerase chain reaction; SNP, single nucleotide polymorphism.

### 3.3. Publication by Country

**Figure 2:**
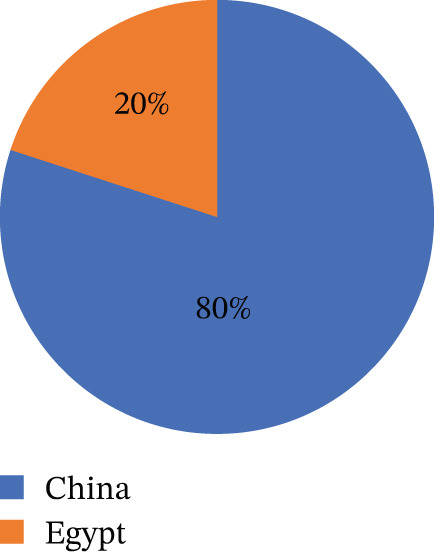
Publication by country.

The countries of origin for included studies are shown in Figure 2. The results indicated that four articles (80%) were published in China [4, 7, 11, 12], whereas one (20%) of the publications was from Egypt [12].

### 3.4. Publication by Year

**Figure 3:**
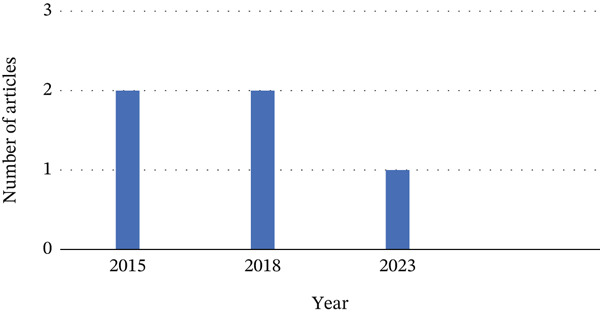
Publication by year.

All the articles included in the systematic review were published between 2015 and 2023 as shown in Figure 3. The results indicated that two articles (*n* = 2) were published in the years 2015 [7, 12], and 2018 [4, 11], and one article (*n* = 1) was published in 2023 [10].

### 3.5. Publication by Journal

**Figure 4:**
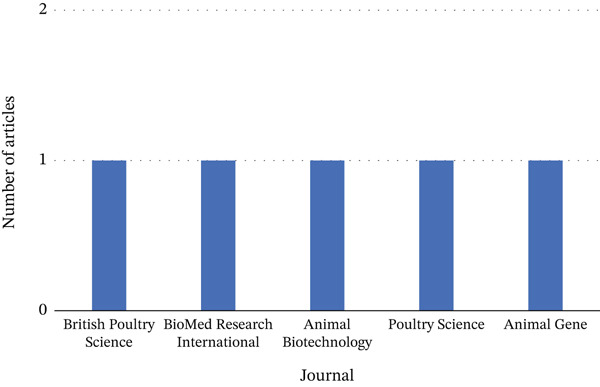
Publication by journal.

The results indicated that all the included studies were published in various journals shown in Figure 4. The results indicated that the following journals had only one publication (*n* = 1) each: *British Poultry Science Journal* [7], *Poultry Science* [10], *BioMed Research International* [4], *Animal Biotechnology* [11], and *Animal Gene* [10].

### 3.6. Distribution of Articles by *GDF-9* Gene Genetic Variation

**Figure 5:**
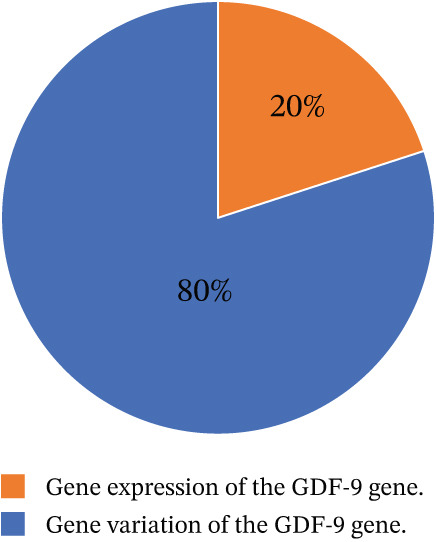
Distribution by *GDF-9* gene genetic variation.

Figure 5 shows the distribution of articles by *GDF-9* gene genetic variation. The results indicate that four articles (*n* = 4) reported genetic variation in the *GDF-9* gene [4, 7, 11, 12], whereas one article (*n* = 1) focused on gene expression in oviduct tissues and its association with reproductive traits [10].

### 3.7. Sequence Analysis

The sequence analysis of the *GDF-9* gene is presented in Table 2. The results indicated that four articles (*n* = 4) reported sequence variations in different chicken breeds [4, 7, 11, 12], whereas one article (*n* = 1) focused on gene expression instead of sequence variation [10].

**Table 2:** Sequence analysis.

Author	Breed	Sequence analysis of the *GDF-9* gene
Huang et al. [7]	Shaobo hens	Variations in coding regions of *GDF-9* and *BMP-15*
Qin et al. [12]	Dagu hens	Novel variants in *GDF-9* and *FOXL2*
Liu et al. [4]	Dongxiang blue‐shelled, Luhua	Exonic mutations in *GDF-9*
Lou et al. [11]	Jinghai Yellow	Base‐replacement mutations in *GDF-9* coding sequence
Habashy and Adomako [10]	Lohman Brown, Golden Sabahia, and White Leghorn	Expression study in oviduct tissues, no mutations reported.

Abbreviations: *BMP-15*, *bone morphogenetic protein 15*; *FOXL2*, *forkhead box L2*; *GDF-9*, g*rowth differentiation factor-9*.

### 3.8. SNPs and Regions

Table 3 shows the polymorphisms of the *GDF-9* gene. The results indicated that four articles (*n* = 4) identified SNPs in various chicken breeds [4, 7, 11, 12].

**Table 3:** Single nucleotide polymorphisms (SNPs) and regions.

Author	Breed	Sample size	Region	SNPs identified
Huang et al. [7]	Shaobo hens	350	Exon 1 (*BMP-15*)	C34T, A111G, C231T
			Exon 2 (*GDF-9*)	G593A, T824C, C896T

Liu et al. [4]	Dongxiang blue‐shelled and Luhua	511	Promoter region	g.17156328G>T, g.17156387C>T, g.17156427A>G
			Exon 1	g.17156703A>C
			Exon 2	g.17158286A>G, g.17158915T>C
			3 ^′^ UTR	g.17159060T>C, g.17159524A>T, g.17159575C>T, g.17159624T>G, g.17159625T>C, g.17159748C>T, g.17159978A>G, g.17160044T>C, g.17160047G>A

Lou et al. [11]	Jinghai Yellow	373	Exon 2 (*GDF-9*)	g.2053G>A, g.2275T>C, g.2338C>T
			3 ^′^ UTR	g.2420T>C

Qin et al. [12]	Dagu hens	360	Exon 2 (*GDF-9*)	G1609T
			Exon 2 (*FOXL2*)	A238G

Abbreviations: 3 ^′^ UTR, 3 ^′^ untranslated region; *BMP-15*, *bone morphogenetic protein 15*; *FOXL2*, *forkhead box L2*; *GDF-9*, *growth differentiation factor-9*; SNP, single nucleotide polymorphism.

### 3.9. Genotypic and Allelic Frequencies

Table 4 presents the genotypic and allelic frequencies of the *GDF-9* gene. The results indicated that three articles (*n* = 4) reported allele and genotype distributions [4, 7, 11, 12].

**Table 4:** Genotypic and allelic frequencies.

Author	Breed(s)	Gene	SNPs	Number	Genotypic frequencies	Allelic frequencies
Huang et al. [7]	Shaobo hens	*BMP-15*	C34T	81	CC: 0.23	C: 0.44
				175	CT: 0.50	T: 0.56
				133	TT: 0.38	
			A111G	49	AA: 0.14	A: 0.46
				207	AG: 0.59	G: 0.54
				94	GG: 0.26	
			C231T	98	C_1_C_1_: 0.28	C_1_: 0.55
				189	C_1_T_1_: 0.54	T_1_: 0.45
				63	T_1_T_1_: 0.18	
		*GDF-9*	G593A	203	GG: 0.58	G: 0.66
				130	GA: 0.37	A: 0.34
				17	AA: 0.05	
			T824C	266	TT: 0.76	T: 0.91
				84	TC: 0.16	C: 0.09
			C896T	46	T_1_T_1_: 0.13	C_1_: 0.77
				74	C_1_T_1_: 0.21	T_1_: 0.23
				230	C_1_C_1_: 0.66	

Liu et al. [4]	Dongxiang blue‐shelled	*GDF-9*	g.17156387C>T	129	CC: 0.46	C: 0.69
				128	CT: 0.46	T: 0.31
				22	TT: 0.08	
			g.17156427A>G	210	AA: 0.75	A: 0.865
				63	AG: 0.23	G: 0.135
				6	GG: 0.02	
			g.17156703A>C	115	AA: 0.41	A: 0.635
				126	AC: 0.45	C: 0.365
				38	GC: 0.14	
	Luhua	*GDF-9*	g.17156387C>T	188	CC: 0.81	C: 0.895
				38	CT: 0.17	T: 0.105
				6	TT: 0.02	
			g.17156427A>G	94	AA: 0.41	A: 0.63
				102	AG: 0.44	G: 0.37
				36	GG: 0.15	
			g.17156703A>C	73	AA: 0.32	A: 0.545
				105	AC: 0.45	C: 0.455
				54	CC: 0.23	

Lou et al. [11]	Jinghai Yellow	*GDF-9*	g.2053G>A	169	GG: 0.45	G: 0.66
				51	AA: 0.14	A: 0.34
				153	AG: 0.41	
			g.2275T>C	153	TT: 0.43	T: 0.66
				41	CC: 0.11	C: 0.34
				167	CT: 0.46	
			g.2338C>T	167	C_1_C_1_: 0.45	C_1_: 0.67
				40	T_1_T_1_: 0.11	T_1_: 0.33
				163	C_1_T_1_: 0.44	
			g.2420T>C	259	T_2_T_2_: 0.70	C_1_: 0.84
				11	C_2_C_2_: 0.03	T_1_: 0.16
				100	C_2_T_2_: 0.27	

Qin et al. [12]	Dagu hens	*GDF-9*	G1609T	231	TT: 0.642	T: 0.821
				129	TC: 0.358	C: 0.179
		*FOXL2*	A238G	246	AA: 0.683	A: 0.842
				114	AB: 0.317	B: 0.158

Abbreviations: *BMP-15*, *bone morphogenetic protein 15*; *FOXL2*, *forkhead box L2*; *GDF-9*, *growth differentiation factor-9*.

### 3.10. Association of Genotypes With Reproduction Traits

The associations of *GDF-9* gene variations with reproductive traits are illustrated in Table 5. The results indicated that four articles (*n* = 4) analyzed genotype–trait associations [4, 7, 11, 12], whereas one article (*n* = 1) reported on gene expression linked to reproduction [10].

**Table 5:** Association of genotypes with reproduction traits.

Author	Breed	Gene	SNPs	Reproduction traits	Genotypes	Significant
Habashy and Adomako [10]	Lohman Brown, Golden Sabahia, and White Leghorn	*GDF-9* (expression)	—	EPR	—	∗
			—	FSH	—	∗
			—	LH	—	∗
			—	E2	—	∗

Huang et al. [7]	Shaobo hens	*BMP-15*	C34T	AFE	CC CT TT	∗
				WFE	CC CT TT	ns
				BWFE	CC CT TT	ns
				EN	CC CT TT	∗
			A111G	AFE	AA AG GG	ns
				WFE	AA AG GG	ns
				BWFE	AA AG GG	ns
				EN	AA AG GG	ns
			C231T	AFE	C_1_C_1_ C_1_T_1_ T_1_T_1_	∗
				WFE	C_1_C_1_ C_1_T_1_ T_1_T_1_	ns
				BWFE	C_1_C_1_ C_1_T_1_ T_1_T_1_	ns
				EN	C_1_C_1_ C_1_T_1_ T_1_T_1_	ns
		*GDF-9*	G593A	AFE	GG GA AA	ns
				WFE	GG GA AA	∗
				BWFE	GG GA AA	ns
				EN	GG GA AA	∗
			T824C	AFE	TT TC	ns
				WFE	TT TC	ns
				BWFE	TT TC	ns
				EN	TT TC	ns
			C896T	AFE	T_1_T_1_ C_1_T_1_ C_1_C_1_	∗
				WFE	T_1_T_1_ C_1_T_1_ C_1_C_1_	∗
				BWFE	T_1_T_1_ C_1_T_1_ C_1_C_1_	ns
				EN	T_1_T_1_ C_1_T_1_ C_1_C_1_	ns

Liu et al. [4]	Dongxiang blue‐shelled and Luhua	*GDF-9*	g.17156387C>T	AFE	CC CT TT	∗
				WFE	CC CT TT	∗
				EWTA	CC CT TT	ns
				EN	CC CT TT	ns
			g.17156427A>G	AFE	AA AG GG	ns
				WFE	AA AG GG	ns
				EWTA	AA AG GG	∗
				EN	AA AG GG	ns
			g.17156703A>C	AFE	AA AC GC	∗
				WFE	AA AC GC	ns
				EWTA	AA AC GC	ns
				EN	AA AC GC	ns

Lou et al. [11]	Jinghai Yellow	*GDF-9*	g.2053G>A	AFE	GG AA AG	ns
				WFE	GG AA AG	ns
				AEWD300	GG AA AG	ns
				END300	GG AA AG	ns
			g.2275T>C	AFE	TT CC CT	ns
				WFE	TT CC CT	ns
				AEWD300	TT CC CT	ns
				END300	TT CC CT	ns
			g.2338C>T	AFE	C_1_C_1_ T_1_T_1_ C_1_T_1_	ns
				WFE	C_1_C_1_ T_1_T_1_ C_1_T_1_	ns
				AEWD300	C_1_C_1_ T_1_T_1_ C_1_T_1_	ns
				END300	C_1_C_1_ T_1_T_1_ C_1_T_1_	ns
			g.2420T>C	AFE	T_2_T_2_ C_2_C_2_ C_2_T_2_	ns
				WFE	T_2_T_2_ C_2_C_2_ C_2_T_2_	ns
				AEWD300	T_2_T_2_ C_2_C_2_ C_2_T_2_	∗∗
				END300	T_2_T_2_ C_2_C_2_ C_2_T_2_	ns

Qin et al. [12]	Dagu hens	*GDF-9*	G1609T	HHEP30	TT TC	∗
				HHEP43	TT TC	∗
				HHEP57	TT TC	∗
				HHEP66	TT TC	∗
				EW30	TT TC	Ns
				EW47	TT TC	∗
		*FOXL2*	A238G	HHEP30	AA AB	∗
				HHEP43	AA AB	∗
				HHEP57	AA AB	∗
				HHEP66	AA AB	∗
				EW30	AA AB	ns
				EW47	AA AB	∗

*Note:* Single asterisk “∗” denotes association and double asterisks “∗∗” mean extremely associated.

Abbreviations: AEWD300, average egg weight at 300 days; AFE, age at first egg; *BMP-15*, *bone morphogenetic protein 15*; BWFE, body weight at first egg; E2, estradiol (a form of estrogen); EN, egg number; END300, egg number at 300 days; EPR, egg production rate; EW30, egg weight at 30 weeks; EW47, egg weight at 47 weeks; EWTA, egg weight at 300 days; *FOXL2*, *forkhead box L2*; FSH, follicle‐stimulating hormone; *GDF-9*, *growth differentiation factor-9*; HHEP30, hen‐housed egg production at 30 weeks; HHEP43, hen‐housed egg production at 43 weeks; HHEP57, hen‐housed egg production at 57 weeks; HHEP66, hen‐housed egg production at 66 weeks; LH, luteinizing hormone; ns, no association; WFE, weight of first egg.

## 4. Discussion

Egg production in chickens is a complex biological process influenced by multiple genes, including the GDF‐9 gene, which plays an essential role in follicular development and ovulation [3, 4]. Understanding the association between SNPs in the GDF‐9 gene and egg production traits is important for improving reproductive efficiency through molecular breeding approaches such as MAS [10, 13]. This systematic review is aimed at evaluating literature on the association between variations of the *GDF-9* gene in chickens and egg characteristics. A total of five peer‐reviewed articles were included in this systematic review based on defined inclusion criteria. The findings revealed several SNPs within the GDF‐9 gene, among which nine were repeatedly reported: g.171515C>T, g.171530A>G, g.171529G>A, g.171554C>T, g.171577A>G, g.171600C>T, g.171541A>G, g.171560T>C, and g.171589C>T. Most of these polymorphisms were significantly associated with egg traits such as age at first egg (AFE), egg number (EN), and EW. [7] identified GDF‐9 polymorphisms (g.171515C>T and g.171530A>G) in Shaobo hens, which were strongly correlated with AFE and EN at 300 days. Similarly, Liu et al. [4] detected four SNPs (g.171577A>G, g.171529G>A, g.171554C>T, and g.171600C>T) in Dongxiang blue‐shelled and Luhua chickens that were associated with reproductive traits, including AFE, WFE, EWTA, EN, and BWTA. Lou et al. [11] further identified SNPs (g.171541A>G, g.171560T>C, and g.171589C>T) in Jinghai Yellow chickens, where certain diplotype combinations showed stronger associations with AFE, EN300, and average EW than individual SNPs. Qin et al. [12] reported novel SNPs (g.171527G>A, g.171589C>T, g.46642995A>G, g.46643104T>C, and g.46643151C>T) in Dagu hens, with specific haplotypes significantly associated with AFE, EN, and EW. Based on our knowledge, this is the first systematic review to summarize findings on the association between GDF‐9 gene SNPs and egg production traits in chickens; therefore, no direct comparison with previous systematic reviews could be made. The findings imply that genetic variants in the GDF‐9 gene may serve as useful molecular markers for selecting layers with superior reproductive performance and egg‐laying efficiency. These results can be applied to the design of genomic and MAS programs aimed at improving egg productivity and overall breeding efficiency in chickens. The contribution of this review to the body of knowledge is the identification of specific GDF‐9 SNPs that are consistently associated with reproductive and egg production traits. These markers provide a valuable foundation for molecular selection and genetic improvement in layer chickens. However, several limitations were noted. Most included studies were conducted in China, which may restrict the applicability of the findings to other chicken populations. In addition, small sample sizes in some studies may have reduced statistical power and affected the strength of associations reported. This systematic review suggests that there is insufficient evidence on the effects of GDF‐9 gene polymorphisms in genetically diverse or indigenous chicken populations. Thus, future studies should validate the identified SNPs using larger and more diverse chicken populations across different environments. Future research should also integrate polymorphism analysis with gene expression profiling to better understand the functional mechanisms of GDF‐9 in egg production. Although several studies have identified associations between GDF‐9 polymorphisms and egg production traits, the precise cellular and molecular mechanisms regulating follicular development remain poorly understood. GDF‐9′s regulatory action within ovarian follicles is anticipated to be very varied among various cell types, such as granulosa cells, theca cells, stromal cells, and oocytes, because it operates via the transforming growth factor‐beta (TGF‐*β*) signaling pathway [14]. This cellular heterogeneity cannot be fully resolved using conventional bulk transcriptomic approaches. To better understand how GDF‐9‐mediated TGF‐*β* signaling controls follicular development and egg production traits in chickens, future research should make use of single‐cell transcriptomic technologies like single‐cell RNA sequencing (scRNA‐seq) to characterize cell‐type–specific signaling dynamics [14, 15]. Future research should therefore move beyond bulk tissue‐level analyses and adopt single‐cell resolution approaches such as scRNA‐seq. This technology enables the characterization of heterogeneous ovarian cell populations, including granulosa cells, theca cells, stromal cells, and oocytes at different developmental stages, thereby providing deeper insight into follicular development and intraovarian signaling pathways [15]. Furthermore, scRNA‐seq can facilitate the identification of cell‐specific expression patterns and signaling interactions associated with GDF‐9 and its downstream pathways during folliculogenesis. Previous studies have demonstrated that GDF‐9 plays a critical role in oocyte–granulosa cell communication and follicular growth regulation [14]. To further dissect the regulatory network of the GDF‐9 pathway, future studies should employ scRNA‐seq to identify cell‐type–specific expression patterns and molecular interactions, as demonstrated in recent high‐resolution biological studies (PMID: 39245652; PMID: 39147128). Integrating traditional genetic variation analyses with emerging single‐cell transcriptomic technologies may represent the next frontier in improving poultry reproductive efficiency and precision breeding strategies.

## 5. Conclusion

In conclusion, the findings of this systematic review demonstrate that genetic variation in the GDF‐9 gene is significantly associated with key egg production traits, including AFE, EN, and EW. These results suggest that GDF‐9 polymorphisms can be used as potential molecular markers to enhance selection accuracy and improve reproductive performance in layer chickens. However, the practical application of GDF‐9 in poultry breeding programs will benefit from a deeper mechanistic understanding of its regulatory functions, particularly through advanced approaches such as single‐cell transcriptomic analysis, which may provide cell‐type–specific insights into reproductive pathways and improve precision breeding strategies.

## Funding

No funding was received for this manuscript.

## Conflicts of Interest

The authors declare no conflicts of interest.

## Data Availability

Data are available upon request.
